# 3D Microstructure Inhibits Mesenchymal Stem Cells Homing to the Site of Liver Cancer Cells on a Microchip

**DOI:** 10.3390/genes8090218

**Published:** 2017-09-01

**Authors:** Xingyuan Yang, Xinyue Xu, Yuan Zhang, Weijia Wen, Xinghua Gao

**Affiliations:** 1 Materials Genome Institute, Shanghai University, Shanghai 200444, China; yangxyshu@163.com (X.Y.); Xuxinyue1229@163.com (X.X.); zhangyuan@shu.edu.cn (Y.Z.); phwen@ust.hk (W.W.); 2 Biomedical Research Institute, Shenzhen Peking University—The Hong Kong University of Science and Technology Medical Center, Shenzhen 518000, China; 3 Department of Physics, The Hong Kong University of Science and Technology, Clear Water Bay, Kowloon 999077, Hong Kong, China

**Keywords:** microchip, mesenchymal stem cells, cell homing, cell co-culture, microstructure

## Abstract

The cell microenvironment consists of multiple types of biophysical and biochemical factors, and represents a complex integrated system that is variable in both time and space. Studies show that changes in biochemical and biophysical factors in cell microenvironments result in significant changes in cellular forms and functions, especially for stem cells. Mesenchymal stem cells (MSCs) are derived from adult stem cells of the mesoderm and play an important role in tissue engineering, regenerative medicine and even cancer therapy. Furthermore, it is found that MSCs can interact with multiple types of tumor cells. The interaction is reflected as two totally different aspects. The negative aspect is that MSCs manifest as tumor-associated fibroblasts and could induce migration of cancer cells and promote tumor formation. On the other hand, MSCs can home to sites of the tumor microenvironment, directionally migrate toward tumor cells and cause tumor cell apoptosis. In this study, we designed and made a simple microfluidic chip for cell co-culture, and studied stem cell homing behavior in the interaction between MSCs and liver cancer cells. Moreover, by etching a three-dimensional microstructure on the base and adding transforming growth factor-β (TGF-β) in the co-culture environment, we studied the impact of biophysical and biochemical factors on stem cell homing behavior, and the causes of such impact.

## 1. Introduction

Derived from adult stem cells of the mesoderm, mesenchymal stem cells (MSCs) mostly exist in the bone marrow, and are important seed cells in tissue engineering [1]. With typical characteristics of pluripotent stem cells, MSCs can be constructed in vitro for subculture, and differentiate toward bones, fat, cartilages or other directions under different inducing conditions [2,3]. With continuous research into MSCs in recent years, it has been found that MSCs can interact with multiple types of tumor cells as the progenitor cells of stromal cells or fibroblasts [4,5]. The interaction is reflected as two totally different aspects [6]. The negative aspect is that MSCs manifest as tumor-associated fibroblasts. As a result, MSCs not only experience phenotypic variation under the inducement of tumor cells but also promote tumor cell invasion and vascularization in surrounding areas, which increases the risks of tumor metastasis. For example, MSCs produce a mutual effect on breast cancer cells [7]. On the other hand, MSCs treat tumor microenvironments as damage areas, directionally migrate toward tumor cells and cause tumor cell apoptosis. This helps inhibit the occurrence and development of tumors, such as oral and hepatic tumors [8]. It is observed that great caution must be exercised in the application of MSCs to tumor treatment. Moreover, extensive laboratory and clinical research is required for sufficient verification in order to exclude potential risks.

Different cell behaviors demonstrated by MSCs toward different tumor cells are closely related to cellular microenvironments. As the sum of external environments that cells survive in, the cell microenvironment consists of multiple types of biophysical and biochemical factors, and represents a complex integrated system that is variable in both time and space [9]. Normally, the cell microenvironment is simply generalized as biochemical factors such as extracellular matrix chemical composition, cytokines and other cells [10], and biophysical factors such as extracellular matrix physical properties [11,12], morphology features [13,14] and biomechanics [15]. Studies show that changes in biochemical and biophysical factors in cell microenvironments result in significant changes in cellular forms and functions. These changes are strongly linked with physiological and pathological processes in the organism, and in particular, have a significant impact on the processes of tumor occurrence and development.

So far, there have been reports on the application of MSCs to the treatment of tumors, such as hepatic and oral tumors. Through in vitro co-culture of MSCs and liver cancer cells (HepG-2 cell), researchers discovered that MSCs have apparent homing behavior, and can suppress HepG-2cell proliferation and induce HepG-2cell apoptosis [16]. Moreover, many studies of signal channels associated with MSCs homing behavior have been conducted by changing biochemical factors in cell microenvironments, such as by adding inhibitors, antagonists or growth factors [17,18]. However, due to limited technological means, the influence of biophysical factors of cell microenvironments on the homing behavior of MSCs is rarely reported.

Based on micro-nanofabrication technology, microfluidic technology is widely applied to biology, chemistry, materials and other relevant fields [19,20]. It helps carry out controllable combinations of multiple cell microenvironment factors to achieve in vitro simulation of the cell microenvironment. Examples of such combinations are concentration gradient [21], cell co-culture [22,23,24], biomechanics [25,26], 3D environment and matrix micro/nanostructures [27,28]. Furthermore, the unique advantage of the microfluidic chip is reflected in its real-time observation of cell behaviors (particularly movement behavior), and in the intuitive display of such biological processes as cancer cell migration and stem cell homing. In this study, we designed and made a simple microfluidic chip for cell co-culture, and studied stem cell homing behavior in the interaction between MSCs and liver cancer cells. Moreover, by etching a three-dimensional microstructure on the base and adding transforming growth factor-β (TGF-β) in the co-culture environment, we studied the impact of biophysical and biochemical factors on stem cell homing behavior, and causes of such impact.

## 2. Materials and Methods

### 2.1. Design and Fabrication of Microfluidic Chip

**Figure 1:**
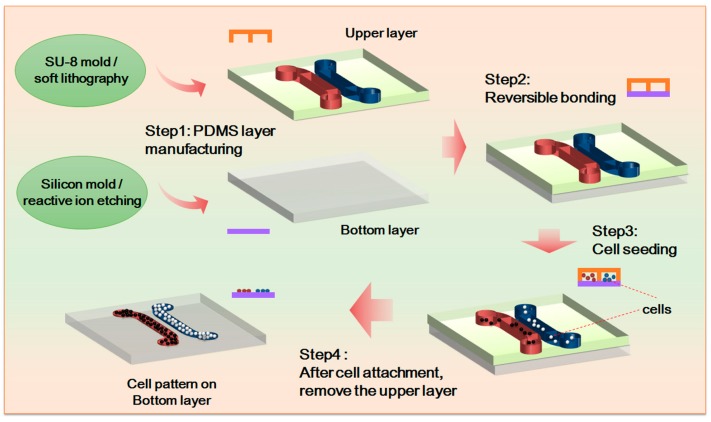
Chip design and experimental process. SU-8 is a high contrast, epoxy based photoresist designed for micromachining and other microelectronic applications, where a thick chemically and thermally stable image is desired. PDMS: polydimethylsiloxane.

In the study, we fabricated the upper channel of the microfluidic chip by means of soft lithography. Made from polydimethylsiloxane (PDMS, Sylgard 184, Dow Corning, Midland, MI, USA), the upper channel includes two independent cell culture channels, which are 1 mm wide and 300 µm deep. The two independent cell culture channels are 500 µm apart so that a gap of non-contact cell co-culture can be formed in the experiment. The gap is also a primary area for observing cell movement. In accordance with the experiments’ purposes, the bottom layer of the chip includes a PDMS 3D microstructure surface and a PDMS flat as the control. The whole chip is fabricated through reversible sealing. For the specific experiment process, see Figure 1.

### 2.2. Design and Fabrication of 3D Microstructure

A 3D form mold was made by adopting photolithographic wet etching. A standard silicon slice with photoresist and a sacrificial layer was purchased. It was then covered by a computer-aided design (CAD)-drawing mask film for the purpose of ultraviolet exposure. After exposure, oxidation etching was performed to etch the sacrificial layer. Ion etching was used to further etch the silicon slice to the desired depth. The optical cement that covered the surface was washed off. Further oxidation etching was carried out to etch the remaining sacrificial layer. After that, a specific structure was obtained.

**Figure 2:**
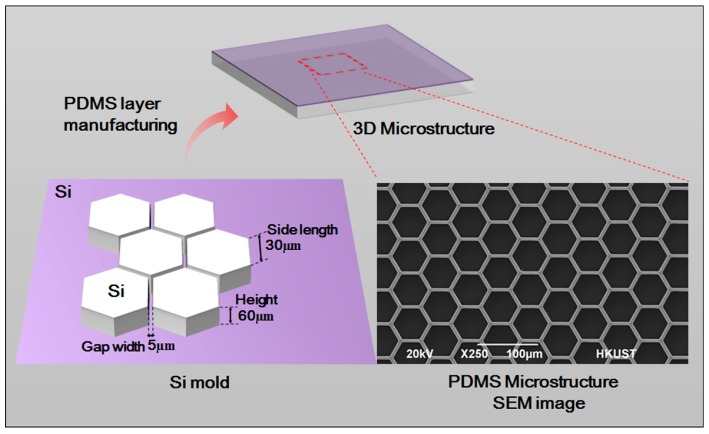
Silicon model design and scanning electron microscope (SEM) image of the PDMS microstructure.

After being placed into absolute ethyl alcohol, soaked and treated by ultrasonic wave, the etched silicon slice was washed with purified water three times, and was thoroughly dried in a 65 °C oven. The mixture of PDMS monomer and initiator (10:1, *v*/*v*) was poured on the fabricated silicon slice board. After bubbles were removed, the board was put in a 65 °C oven for solidification. The fabricated PDMS 3D microstructure scanning electron microscope (SEM) is shown as in Figure 2. The microstructure is 60 µm high and has a hexagon frame with side length of 30 µm. The gap width is 5 µm. The neat and even structure with smooth surface can be used in subsequent experiments.

### 2.3. Cell Culture and Seeding

In the experiment, MSCs and liver cancer cell (HepG-2) were co-cultured, and mouse embryo fibroblast NIH-3T3 was chosen as the control cell. The culture medium for MSCs is medium α-MEM (Minimum Essential Medium α, Gibco, Gaithersburg, MD, USA) that contains 10% fetal bovine serum (FBS, Hyclone, Logan, UT, USA). The culture medium for liver cancer cell (HepG-2) and cell NIH-3T3 is high-glucose medium DMEM (Dulbecco’s Modified Eagle’s Medium, Gibco, Gaithersburg, MD, USA) that contains 10% FBS. High-glucose medium DMEM containing 2% FBS was chosen and used to suppress cell proliferation in the process of chip co-culture.

Out of experiment needs, we inoculated HepG-2 or NIH-3T3 cell suspension of around 10 µL 2 × 10^5^/mL to the channel on one side of the chip; and MSCs cell suspension of about 10 µL 1 × 10^5^/mL to the channel on the other side of the chip. After 24 h, when all the cells adhered, the upper chip was taken off, and cell patterns from cell non-contact co-culture were formed. Co-culture medium was added, and cell migration was observed at regular intervals.

### 2.4. Cell Staining and Observation

To check whether tumor cell metastasis or stem cell homing occurs in the process of HepG-2 and MSCs co-culture, we used a laser scanning confocal microscope and live cell station to observe cell co-culture in real time. Then, we performed regular observation with an inverted fluorescence microscope for the purpose of image acquisition.

In the experiment, phalloidine (Alexa Fluor 488 Phallidin) from the company Life Invitrogen (Carlsbad, CA, USA) was used for fluorescent characterization of cytoskeleton F-actin. The detailed procedure was as follows: add HepG-2 cell suspension of around 1 mL 1 × 10^5^/mL on the 3D microstructure surface; remove the culture medium after cells are adhered to the wall for 24 h, and wash the cells with phosphate buffer solution (PBS) three times; process the cells with 0.1%Triton-X 100 (Sigma, St. Louis, MO, USA) for 5 min, and wash them with PBS twice; add the working solution Alexa Fluor 488 Phallidin into the cells, and leave aside for 20 min at room temperature; after that, wash the cells with PBS, and 4',6-Diamidino-2-Phenylindole (DAPI) (Life Invitrogen, Carlsbad, CA, USA) stain cell nucleuses before taking images. The working solution Alexa Fluor 488 Phallidin is a mixture of 6.6 micromole/liter methyl alcohol phalloidine mother solution of 5 microliters and 1% BSA (Sigma, St. Louis, MO, USA) PBS solution of 200 µL.

In the experiment, immunofluorescent staining was adopted to detect the proteins associated with epithelial-mesenchymal transition (EMT), including vimentin (R&D Systems, Minneapolis, MN, USA) and E-cadherin (E-cad, R&D Systems). The staining method is as follows: carefully wash post-experiment cells with 4 °C PBS solution after cell treatment for 48 h, and fixate them with 4% paraformaldehyde for 20 min; add 0.1%Triton-X 100 to process the cells for 10 min (this step is skipped in E-cad straining); add goat serum working solution to the cells and keep them sealed for 1 h at room temperature; add primary antibody working solution diluted by the proportion 1:100 (rabbit anti-mouse vimentin; rabbit anti-mouse E-cad) into the cells, and leave aside for a whole night in a 4 °C fridge; wash the cells with PBS and add second antibody working solution (goat anti-rabbit IgG) marked by fluorescein isothiocyanate (FITC); incubate for 1 h at room temperature; add DAPI working solution and perform room-temperature incubation for 30 min; wash the cells with PBS three times, and then cell images can be taken.

### 2.5. Data Statistics

Cells were visualized using an Olympus fluorescence microscope (Olympus IX 71, Tokyo, Japan). The cell images collected were processed by the software Image-Pro (Media Cybernetics, Rockville, MD, USA) for data analysis, covering data such as cell counts and fluorescent areas. Student’s *t*-test was conducted with the standard deviation in experimental data greater than or equal to 3.

## 3. Results

### 3.1. Interaction between Hepatoma Cells and MSCs

**Figure 3:**
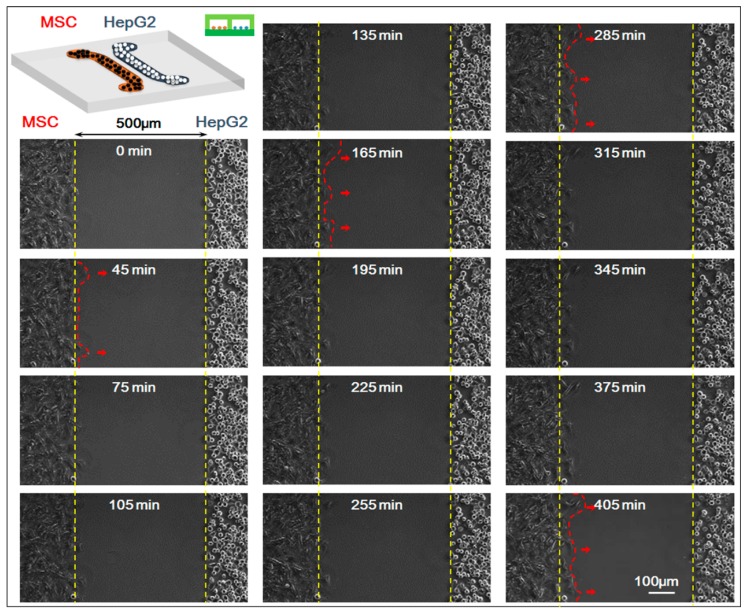
Time-lapse images of interactions between mesenchymal stem cells (MSCs) and HepG-2 cells. The starting time (0 min) indicated the first 5 min after the upper chip was removed and after that, a picture was taken every 15 min; a total of 28 pictures (405 min) were taken. Fourteen pictures were shown. Bar is 100 µm.

In the experiment, we studied the co-culture process of HepG-2 and MSCs with the real-time monitoring system of the microscopic living cell workstation. We compared the movement of HepG-2 and MSCs in the 0–405 min when the cells were in non-contact co-culture status (the starting time 0 min indicated the first 5 min after the upper chip was removed and after that, a picture was taken every 15 min; a total of 28 pictures were taken). Results showed that HepG-2 hardly moved while MSCs migrated clearly toward HepG-2 cells, which was the homing behavior of MSCs. For specific results of the experiment, see Figure 3.

**Figure 4:**
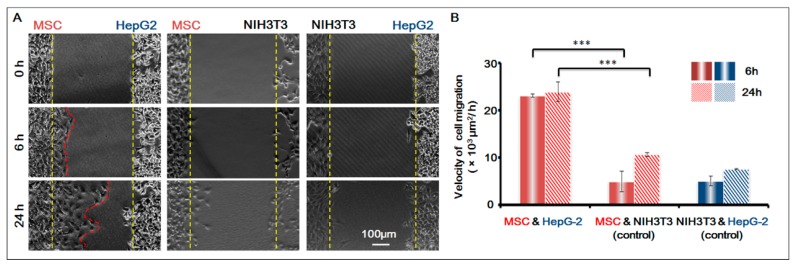
MSCs homing to the sites of HepG-2 cells. (**A**) Cell images under different conditions. Bar is 100 µm; (**B**) Histogram of cell migration velocity of MSCs (MSC and HepG-2 group; MSC and NIH-3T3 group) and HepG-2 cells (NIH-3T3 and HepG-2 Group) in 6 h and 24 h. *p* < 0.005, ***. *n* = 3.

To further confirm that the homing behavior of MSCs occurred, we used NIH-3T3 as the control cell, and studied the co-culture of three groups of cells respectively: MSCs and HepG-2; MSCs and NIH-3T3; HepG-2 and NIH-3T3. Based on the migration area, we calculated the migration speed of the cells in 6 h and 24 h respectively. For results, see Figure 4. It was obvious that before the gap area was completely filled, MSCs showed apparent homing behavior toward the direction of HepG-2 and their speed of migration was basically the same; under the same conditions, however, MSCs did not migrate towards the control cell NIH-3T3. The result was conclusive evidence that MSCs had obvious homing behavior toward HepG-2.

### 3.2. Homing Behavior of MSCs under Different Conditions

**Figure 5:**
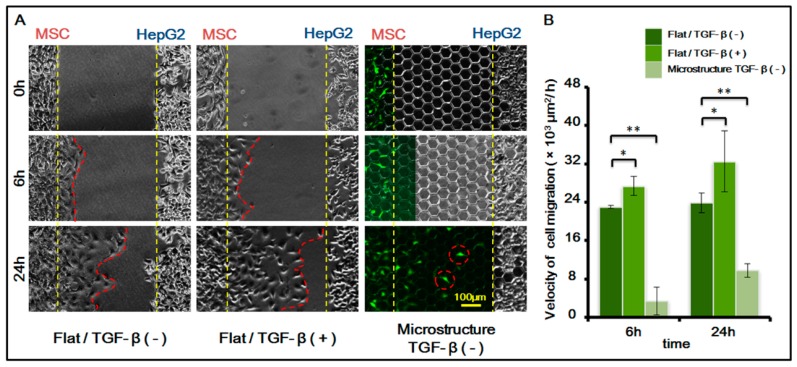
MSCs homing to the sites of HepG-2 cells under different biochemical and biophysical conditions. (**A**) Cell images under different conditions. MSCs transfected with green fluorescent protein (GFP, green). TGF-β is transforming growth factor-beta. (1 ng/mL). Bar is 100 µm; (**B**) Histogram of cell migration velocity of MSCs under different conditions in 6 h and 24 h. *p* < 0.05, *; *p* < 0.01, **. *n* = 3.

After confirming the existence of MSC homing behavior toward HepG-2, we further studied MSC homing in the following two circumstances: on the surface of a three-dimensional micro-structure and in the presence of chemical inducing substance TGF-β with 1 ng/mL. For study results, see Figure 5.

The results indicated that the migration speed of MSC homing becomes markedly higher after chemical inducing substance TGF-β is added. TGF-β is an important growth factor that has high expression in multiple tumor cells and adjusts cell growth and differentiation. It is a corroborated protein associated with MSC homing. In this experiment, we added TGF-β to verify its induction to MSC homing. The experiment also indirectly indicated that MSCs demonstrate homing behavior toward HepG-2, which might be related to the fact that TGF-β can be secreted by HepG-2 itself.

Furthermore, results also showed that the presence of a three-dimensional micro-structure suppresses MSC homing. The suppression might be caused by the following two factors: (1) the physical blocking function of the three-dimensional micro-structure itself inhibits the movement of MSCs, therefore suppressing its homing behavior; (2) the three-dimensional micro-structure itself might affect the secretion of TGF-β by HepG-2, weakening the role of TGF-β in inducing MSC homing.

### 3.3. Impact of Three-Dimensional Micro-Structure on HepG-2 Cell Phenotype

We analyzed the aforementioned two reasons for the three-dimensional micro-structure’s suppression of MSC homing, and first studied whether the three-dimensional micro-structure affects HepG-2 in secreting TGF-β.

**Figure 6:**
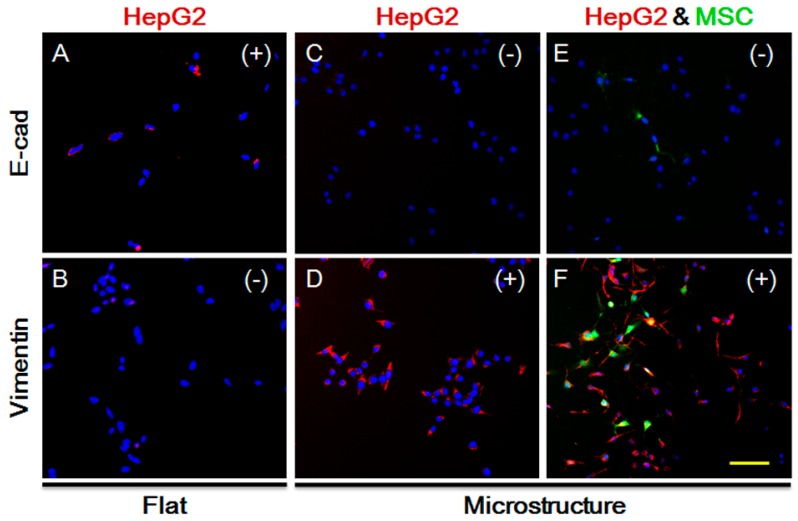
Expression of E-cadherin and Vimentin in HepG-2 cells under different conditions after 48 h. (**A**) Expression of E-cadherin in HepG-2 cells on flat substrate; (**B**) Expression of Vimentin in HepG-2 cells on flat substrate; (**C**) Expression of E-cadherin in HepG-2 cells on 3D microstructure; (**D**) Expression of E-cadherin in HepG-2 cells on 3D microstructure; (**E**) Expression of E-cadherin in HepG-2 cells on 3D microstructure with MSCs; (**F**) Expression of E-cadherin in HepG-2 cells on 3D microstructure with MSCs; Green, MSC cells. Red, expression positive HepG-2 cells (E-cadherin or Vimentin). Blue, cell nucleus. (+): positive; (−): negative. Bar is 100 µm.

TGF-β not only impacts MSC homing, but is also closely related to the EMT processes of tumor cells, as indicated by extensive studies. The long-term effect of TGF-β leads to increased expression of vimentin in epithelial cells, but lower expression of E-cadherin, causing occurrence of EMT. We explored the expression of HepG-2 vimentin and E-cadherin on the plane. As shown in Figure 6A,B, vimentin expression is negative while E-cadherin expression is positive, and the cells show no sign of EMT. Under the same conditions and in the presence of the three-dimensional microstructure, protein expression is changed, as shown in Figure 6C,D. Vimentin expression becomes positive while E-cadherin expression is negative. Moreover, we co-cultured MSCs and HepG-2, and detected the protein expression of HepG-2. As demonstrated in Figure 6E,F, vimentin expression is increased significantly, indicating that HepG-2 tends to undergo EMT on the surface of the micro-structure. It also suggests that secretion of TGF-β by HepG-2 does not decline due to the impact of the micro-structure, but might increase instead. Therefore, the influence of the three-dimensional micro-structure on the secretion of TGF-β by HepG-2 is not the reason for the suppression of MSC homing.

### 3.4. Impact of Three-Dimensional Micro-Structure on the Morphology of MSCs

The three-dimensional micro-structure itself does not cause a decrease in TGF-β secreted by HepG-2 and weaken MSC homing. So, its physical blocking function is highly likely to be the main cause of the suppression of MSC homing.

**Figure 7:**
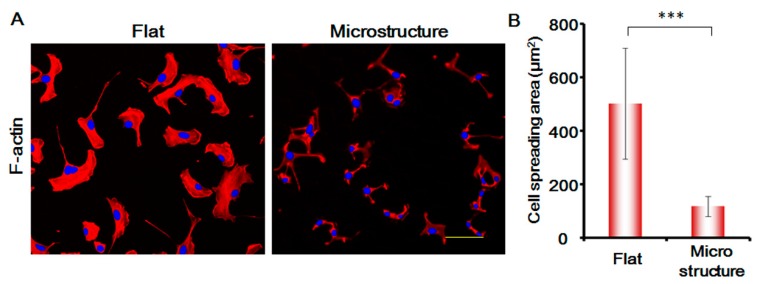
(**A**) Expression of F-actin in MSC cells under different conditions after 24 h. Red, F-actin. Blue, cell nucleus. Bar is 100 µm; (**B**) Histogram of the cell spreading area. *p* < 0.005, ***. *n* = 3.

In this study, we stained the cytoskeletal protein F-actin of MSCs, and analyzed the changes in the morphology of MSCs. As shown in Figure 7, the spreading area of MSCs on the surface of micro-structure decreases enormously and is only around one-third of the flat spreading area. The areas of the cell nucleus are not changed significantly. The morphology of the cells is greatly suppressed, which affects the migration speed of the cells to a large degree. This shows that the physical blocking function of the three-dimensional micro-structure is the primary factor responsible for suppressing MSCs.

## 4. Conclusions

Adopting micro-nanofabrication technology and microfluidic technology, we designed and prepared three-dimensional micro-structure surfaces and achieved non-contact co-culture of MSCs and hepatoma cells by means of cell patterning technology. It was found that MSCs showed homing behavior under the action of hepatoma cells. Moreover, based on characterization of the cell spreading area and EMT-related protein expression, it was discovered that the three-dimensional micro-structure might suppress the homing behavior of MSCs and the suppression was achieved mainly through the physical blocking function of the microstructure.

## References

[1] Chamberlain G., Fox J., Ashton B., Middleton J. (2007). Concise review: Mesenchymal stem cells: Their phenotype, differentiation capacity, immunological features, and potential for homing. Stem Cells.

[2] Pittenger M.F., Mackay A.M., Beck S.C., Jaiswal R.K., Douglas R., Mosca J.D., Moorman M.A., Simonetti D.W., Craig S., Marshak D.R. (1999). Multilineage potential of adult human mesenchymal stem cells. Science.

[3] Jiang Y., Jahagirdar B.N., Reinhardt R.L., Schwartz R.E., Keene C.D., Ortiz-Gonzalez X.R., Reyes M., Lenvik T., Lund T., Blackstad M. (2002). Pluripotency of mesenchymal stem cells derived from adult marrow. Nature.

[4] Karp J.M., Teo G.S.L. (2009). Mesenchymal stem cell homing: the devil is in the details. Cell Stem Cell.

[5] Spaeth E., Klopp A., Dembinski J., Andreeff M., Marini F. (2008). Inflammation and tumor microenvironments: defining the migratory itinerary of mesenchymal stem cells. Gene Ther..

[6] Karaoz E., Akpinar B. (2013). Filling the Gap in the Relationship between Cancer and Stem Cells. Stem Cells: Current Challenges and New Directions.

[7] Karnoub A.E., Dash A.B., Vo A.P., Sullivan A., Brooks M.W., Bell G.W., Richardson A.L., Polyak K., Tubo R., Weinberget R.A. (2007). Mesenchymal stem cells within tumour stroma promote breast cancer metastasis. Nature.

[8] Ma H., Zhang M., Qin J. (2012). Probing the role of mesenchymal stem cells in salivary gland cancer on biomimetic microdevices. Integr. Biol..

[9] Park J.Y., Takayama S., Lee S.H. (2010). Regulating microenvironmental stimuli for stem cells and cancer cells using microsystems. Integr. Biol..

[10] Mantovani A., Allavena P., Sica A., Balkwillet F. (2008). Cancer-related inflammation. Nature.

[11] Engler A.J., Sen S., Sweeney H.L., Discheret D.E. (2006). Matrix elasticity directs stem cell lineage specification. Cell.

[12] Guilak F., Cohen D.M., Estes B.T., Gimble J.M., Liedtke W., Chen C.S. (2009). Control of stem cell fate by physical interactions with the extracellular matrix. Cell Stem Cell.

[13] Nguyen A.T., Sathe S.R., Yim E.K.F. (2016). From nano to micro: topographical scale and its impact on cell adhesion, morphology and contact guidance. J. Phys. Condens. Matter.

[14] Théry M. (2010). Micropatterning as a tool to decipher cell morphogenesis and functions. J. Cell Sci..

[15] Discher D., Dong C., Fredberg J.J., Guilak F., Ingber D., Janmey P., Kamm R.D., Schmid-Schönbein G.W., Weinbaumet S. (2009). Biomechanics: cell research and applications for the next decade. Ann. Biomed. Eng..

[16] Qiao L., Xu Z., Zhao T., Zhao Z., Shi M., Zhao R.C., Ye L., Zhang X. (2008). Suppression of tumorigenesis by human mesenchymal stem cells in a hepatoma model. Cell Res..

[17] Hou L., Wang X., Zhou Y., Ma H., Wang Z., He J., Hu H., Guan W., Ma Y. (2014). Inhibitory effect and mechanism of mesenchymal stem cells on liver cancer cells. Tumor Biol..

[18] Chaturvedi P., Gilkes D.M., Wong C.C.L., Kshitiz, Luo W., Zhang H., Wei H., Schito N.T.L., Levchenko A., Semenza G.L. (2013). Hypoxia-inducible factor–dependent breast cancer–mesenchymal stem cell bidirectional signaling promotes metastasis. J. Clin. Investig..

[19] Gupta K., Kim D.H., Ellison D., Smith C., Kundu A., Tuan J., Suhc K.Y., Levchenko A. (2010). Lab-on-a-chip devices as an emerging platform for stem cell biology. Lab Chip.

[20] Ma H., Xu H., Qin J. (2013). Biomimetic tumor microenvironment on a microfluidic platform. Biomicrofluidics.

[21] Eribol P., Uguz A.K., Ulgen K.O. (2016). Screening applications in drug discovery based on microfluidic technology. Biomicrofluidics.

[22] An Y., Ma C., Tian C., Zhao L., Pang L., Tu Q., Xu J., Wang J. (2015). On-chip assay of the effect of topographical microenvironment on cell growth and cell-cell interactions during wound healing. Biomicrofluidics.

[23] Wang L., Tao T., Su W., Yu H., Yu Y., Qin J. (2017). A disease model of diabetic nephropathy in a glomerulus-on-a-chip microdevice. Lab Chip.

[24] Xie Y., Zhang W., Wang L., Sun K., Sun Y., Jiang X. (2011). A Microchip-Based Model Wound with Multiple Types of Cells. Lab Chip.

[25] Gao X., Zhang X., Xu H., Zhou B., Wen W., Qin J. (2014). Regulation of cell migration and osteogenic differentiation in mesenchymal stem cells under extremely low fluidic shear stress. Biomicrofluidics.

[26] Gao X., Zhang X., Tong H., Lin B., Qin J. (2011). A simple elastic membrane-based microfluidic chip for the proliferation and differentiation of mesenchymal stem cells under tensile stress. Electrophoresis.

[27] Gao X., Chau Y.Y., Xie J., Wan J., Ren Y., Qin J., Wen W. (2015). Regulating cell behaviors on micropillar topographies affected by interfacial energy. RSC Adv..

[28] Zhou B., Gao X., Wang C., Ye Z., Gao Y., Xie J., Wu X., Wen W. (2015). Functionalized PDMS with versatile and scalable surface roughness gradients for cell culture. ACS Appl. Mater. Interfaces.

